# Effects of pyrrolidine dithiocarbamate on proliferation and nuclear factor-κB activity in autosomal dominant polycystic kidney disease cells

**DOI:** 10.1186/s12882-015-0193-3

**Published:** 2015-12-15

**Authors:** Michelle H. T. Ta, David Liuwantara, Gopala K. Rangan

**Affiliations:** Centre for Transplant and Renal Research, Level 5, The Westmead Institute for Medical Research, University of Sydney, 176 Hawkesbury Rd, Westmead, NSW 2145 Australia

**Keywords:** Nuclear factor-κB (NF-κB), Polycystic kidney disease, Proliferation, Pyrrolidine dithiocarbamate (PDTC), Tumor necrosis factor (TNF)-α

## Abstract

**Background:**

Pyrrolidine dithiocarbamate (PDTC) reduces renal cyst growth in a rodent model of polycystic kidney disease (PKD) but the mechanism of action is not clear. Here, we investigated the hypothesis that PDTC reduces the proliferation of cystic epithelial cells *in vitro* in a nuclear factor (NF)-κB-dependent manner.

**Methods:**

Immortalized autosomal dominant PKD (ADPKD) cells that are heterozygous (WT9-7) and homozygous (WT-9-12) for a truncating *Pkd1* mutation, and immortalized normal human tubular cells (HK-2), were exposed to NF-κB-inducing agents with or without PDTC. Cell proliferation and apoptosis were assessed by bromodeoxyuridine assay and Annexin V flow cytometry, respectively. NF-κB activity was assessed by luciferase reporter assay and western blotting for nuclear p65, p50, and RelB subunits and cytoplasmic phosphorylated-IκBα.

**Results:**

Serum-induced proliferation was similar in all cell lines over 72 h. PDTC demonstrated anti-proliferative effects that were delayed in ADPKD cells compared to HK-2. Basal NF-κB-dependent luciferase reporter activity was lower in ADPKD cells compared to normal cells. Classical NF-κB stimulants, lipopolysaccharide (LPS) and tumor necrosis factor (TNF)-α, increased NF-κB luciferase activity in HK-2, whereas in PKD cell lines, NF-κB activity was only induced by TNF-α. However, neither stimulant altered proliferation in any cell line. PDTC reduced TNF-α-stimulated NF-κB activity in HK-2 only.

**Conclusions:**

PDTC reduced proliferation in ADPKD cells but did not consistently alter NF-κB activation, suggesting that other signalling pathways are likely to be involved in its ability to attenuate renal cyst growth *in vivo.*

**Electronic supplementary material:**

The online version of this article (doi:10.1186/s12882-015-0193-3) contains supplementary material, which is available to authorized users.

## Background

Polycystic kidney diseases (PKD) are a group of genetically inherited disorders involving the formation of multiple renal cysts [1, 2]. Autosomal Dominant PKD (ADPKD) arises due to mutations in *PKD1* and/or *PKD2* [3, 4] and is characterized by the onset of symptoms in adulthood [2]. In Autosomal Recessive PKD (ARPKD), the mutation of *PKHD1* usually causes lethality during fetal life or in early childhood [2, 5]. Renal failure is one of the leading causes of mortality in PKD, and as there are no specific therapies available, eventually dialysis or renal transplantation is required [1].

The key histological features of PKD are the proliferation and dedifferentiation of cystic epithelial cells (CECs) accompanied by interstitial inflammation and fibrosis [1, 6], and apoptosis [7–9]. Recent data suggest that the nuclear factor (NF)-κB system, a key controller of inflammation and apoptosis [10], is up-regulated in experimental models of PKD [11, 12]. The use of small interfering RNA to overexpress or deplete the protein products of *PKD1* or *PKHD1 in vitro*, resulted in up-regulated NF-κB activity compared to control cells [13, 14]. Increased luciferase NF-κB activity and expression of phosphorylated p65 have been identified in mouse *Pkd1*^*−/−*^ cells compared to wild-type cells [11]. We also previously identified an activated NF-κB protein, phosphorylated p105, in the CECs of the Lewis Polycystic Kidney (LPK) rat (a *NEK8/NPHP9* ortholog phenotypically resembling human ARPKD) [15–17]. Notably, inhibitors of NF-κB modify aberrant apoptosis in mutant PKD cells [13] and decrease cyst area in *Pkd1*^*−/−*^ mouse kidney explants [11].

Pyrrolidine dithiocarbamate (PDTC) is a well-known inhibitor of NF-κB activation capable of decreasing the expression of inflammatory genes, including chemokine (C-C motif) ligand 2 *(CCL2)*, in rat tubular epithelial and mesangial cells [18, 19]. PDTC has known anti-proliferative effects on renal cancer cells [20] and vascular smooth muscle cells [21]. The compound has demonstrated divergent effects on apoptosis, increasing apoptosis in renal cell carcinoma cells [20], but decreasing ischemia-induced and cadmium-induced apoptosis in renal tubular cells [22, 23]. We previously demonstrated that in the LPK rat, chronic administration of PDTC for 7 weeks attenuated kidney enlargement by 25 %, as determined by MRI-assessed total kidney volume and kidney weight [16]. This was associated with a reduction in three dimensional cyst volume but without any changes in interstitial disease or cell proliferation.

The aim of the current study was to determine if PDTC alters the proliferation and survival of CECs *in vitro*. We utilized HK-2 cells (immortalized cells derived from proximal tubules of normal human kidney cortex [24]) and WT9-7 and WT9-12 cells (two immortalized cell lines originally derived from a human ADPKD kidney [25]). Mutational analysis has found that WT9-7 cells are heterozygous for a truncating *PKD1* mutation (Q2556X), while WT9-12 cells are homozygous for this mutant allele [26]. The two cell lines are thought to exemplify the “two-hit hypothesis”, which suggests that while all cells of an ADPKD patient originally possess one mutated and one normal allele, environmentally acquired injury causes a somatic mutation in the normal allele, thereby initiating cyst formation [27]. We therefore utilized the WT9-7 and WT9-12 cell lines as a means of comparing the effects of PDTC on PKD cells that are heterozygous and homozygous for a *PKD1* mutation. We hypothesized that PDTC reduces the proliferation of ADPKD cells and also decreases NF-κB activity in these cells.

## Methods

### Cell culture

All cell lines were obtained from the American Type Culture Collection (ATCC, Manassas, VA) in July 2014. We utilized HK-2 cells (immortalized cells derived from proximal tubules of normal human kidney cortex [24], CRL-2190, Lot no. 61218770, ATCC) and WT9-7 and WT9-12 cells (two immortalized cell lines originally derived from a human ADPKD kidney [25], CRL-2830, Lot no. 58737172, and CRL-2833, Lot no. 60336584, ATCC). Both PKD cell lines were derived from the same kidney cortex, however the WT9-7 cells originated from a non-dilated tubule and possess proximal tubular characteristics, whereas the WT9-12 cells originated from a dilated (cystic) tubule and have both proximal and distal characteristics [25]. The WT9-7 cells are heterozygous for a truncating *PKD1* mutation (Q2556X) and possess the full-length form of polycystin-1 (the gene product of *PKD1*), whereas the WT9-12 cells are homozygous for the Q2556X mutation and express polycystin-1 only in its truncated form [26]. The WT9-7 and WT9-12 cell lines were immortalized by transformation with wild type adeno-simian (SV)40 virus large T antigen [25], which inhibits p53 activity and mediates the progression of the cell cycle into the S-phase [28]. The HK-2 cells were derived from proximal tubular cells of a normal human kidney [24] and were immortalized by human papilloma virus (HPV 16) E6 and E7 genes, which inhibit the pro-apoptotic proteins, p53 and retinoblastoma, respectively [29]. HK-2 cells were cultured in a 1:1 ratio of DMEM and F12, 10 % fetal bovine serum (FBS), with penicillin and streptomycin. WT9 cells were cultured in DMEM (3.7 g/mL sodium bicarbonate), 10 % FBS, with penicillin and streptomycin. All cultures were maintained at 37 °C, 5 % CO_2_.

### Cell viability assay

For all cell viability and proliferation assays, HK-2 and WT9-12 cells were seeded at 5 x 10^3^ cells/well in a 96-well plate, and cultured for 24 h prior to the addition of PDTC. PDTC (Sigma-Aldrich, St. Louis, MO) was dissolved in media and filter-sterilized. For assessment of viability, cells were incubated with vehicle control, 5 μM, 20 μM, or 50 μM PDTC for 6, 24, or 48 h. At the end of each timepoint, the MTT assay (11465007001, Roche Diagnostics, Mannheim, Germany) was performed according to manufacturer’s instructions. Absorbance was measured at 570 nm (reference 750 nm). Cell viability was calculated by 100 x (Absorbance of Sample/Average absorbance of untreated control) for the respective cell line.

### Assessment of proliferation

Cell proliferation was assessed using the bromodeoxyuridine (BrdU) assay (11647229001, Roche) according to manufacturer’s instructions. In the first series of experiments, cells were incubated with 20 μM PDTC for 4, 8 or 24 h. In a separate series of experiments, lipopolysaccharide (LPS, 10ug/ml, L2630, from *E.coli*, Sigma-Aldrich) or tumor necrosis factor-α (TNF-α, 20 ng/ml, human recombinant, #130-094-020, Miltenyi Biotec, Bergisch Gladbach, Germany) was added to the cells for 4 h or 24 h. The concentrations of LPS and TNF-α were based on doses that stimulated NF-κB activity in HK-2 cells in previous studies [30–32]. Four hours before the end of each timepoint, BrdU was added to a final concentration of 10 μM. Absorbance was measured at 450 nm (reference 620 nm). Cell proliferation (%) was calculated as 100 x Absorbance/Average absorbance of the vehicle group, of each experiment.

### Assessment of apoptosis

Cells were seeded in 100 mm dishes and grown for 24 h. Cells were then treated with vehicle, 20 μM PDTC, 200 μM PDTC, or 5 μM camptothecin. After 24 h of treatment, cells were harvested by trypsinization, resuspended at 10^6^ cells/ml in 1X Annexin V Binding Buffer (BD Biosciences, Franklin Lakes, NJ), and 100 μl (10^5^ cells) were added to each FACS tube. Cells were stained with 7-AAD (5 μl/100 μl, #559925, BD Biosciences, to assess cell death), and APC-conjugated Annexin V (5 μl/100 μl, #550474, BD Biosciences, to assess apoptosis) for 15 min and analyzed on BD FACSCantoII. As positive controls for apoptosis and cell death, cells were incubated for 24 h with 5 μM camptothecin (Sigma-Aldrich) [33, 34] or 200 μM PDTC, respectively. FACS data were acquired using FACSDiva (v6.1.3, 2009, BD Biosciences) and analyzed using FlowJo software (v10.0.7r2, Tree Star, Ashland, OR) and the percentage of APC+ cells obtained.

### Luciferase-reporter NF-κB assay

Cells were seeded at 2 x 10^5^ cells per well in a 6-well plate and grown for 24 h. Cells were transfected with 0.4 μg/well of luciferase reporter plasmid 3 × NF-κB(IC)tk-LUC containing 3 repeating NF-κB promoter sequences [35–37] (a kind gift of Dr. Scott Read, Storr Liver Unit, The Westmead Institute for Medical Research , Australia). As a control for transfection efficiency, cells were co-transfected with 0.5 μg/well of pEGFP-N1 N-terminal protein fusion vector containing a human cytomegalovirus (hCMV) promoter (GenBank Accession #U55762, Clontech Laboratories, Palo Alto, CA). Transfection was performed in a final volume of 1 mL media, 10 % FBS, with a 3 μL:1 μg ratio of FugeneHD (Promega Corporation, Madison, WI) to plasmid. Four hours post-transfection an additional 1 mL of media was added. Twenty hours post-transfection, the medium was changed and cells were treated with either vehicle, LPS (10 μg/mL) or TNF-α (20 ng/ml) for a further 4 h. In a separate set of experiments, cells were pre-incubated with 20 μM PDTC for 1 h prior to incubation with LPS and TNF-α. Cells were washed with PBS, lysed using Cell Culture Lysis Reagent (Promega) and assayed in a Victor plate reader (PerkinElmer, Waltham, MA) using Luciferase Reagent (Promega) according to manufacturer’s instructions. Luciferase activity was normalized to GFP fluorescence levels.

### Western blot

For western blots, cells were seeded at 1.2×10^6^ cells per 100 mm dish. Twenty hours post-seeding, media was changed and cells were pre-incubated with 20 μM PDTC for 1 h, then were treated with vehicle, LPS or TNF-α for a further 4 h. Cells were trypsinized and nuclear extraction performed using the NE-PER kit (Thermo Fisher Scientific, Waltham, MA) according to manufacturer’s instructions. Nuclear and cytosolic extracts were stored at −80 °C. Protein concentration of the extracts was assessed using the DC Protein Assay (Bio-Rad Laboratories, Hercules, CA). Whole cell lysates were obtained using RIPA buffer (Thermo Fisher) according to manufacturer’s instructions. For western blotting, proteins were electrophoresed on 4–15 % Mini-PROTEAN stain-free TGX gels and semi-dry transferred to PVDF membranes (Bio-Rad). Membranes were blocked with 5 % BSA then incubated with antibodies for toll-like receptor 4 (TLR4, 1:1000, ab22048, Abcam, Cambridge, UK), p50/105 (1:1000, ab7971, Abcam), p65 (1:1000, #8242, Cell Signaling Technology, Danvers, MA), RelB (1:1000, #4922, Cell Signaling), β-actin (1:1000, #4970, Cell Signaling), phosphorylated-IκBα (p-IκBα, 1:1000, #9246, Cell Signaling), IκBα (1:1000, #4814, Cell Signaling), and GAPDH (1:1000, #5174, Cell Signaling) overnight at 4 °C. Antibodies against p100 (ab6549, Abcam) and p52 (#4882, Cell Signaling) were also tested but yielded no signal by immunoblotting. Secondary antibodies (anti-rabbit IgG, 1:10,000, A0545; anti-mouse, 1:10,000, A6782, Sigma-Aldrich) were applied for 1 h at room temperature. Blots were developed using SuperSignal West Pico and Femto chemiluminescent substrates (Thermo Fisher), and imaged using the Chemidoc MP system (Bio-Rad). Densitometry was quantified using ImageJ (v1.47, National Institutes of Health, USA) and normalized using β-actin (for nuclear extracts) or GAPDH (for cytoplasmic and whole cell extracts). Phosphorylated IκBα was normalized against total IκBα.

### Statistical analyses

Data are expressed as mean ± standard deviation. Statistical analysis was performed using an independent *t*-test (or Mann–Whitney *U* test with non-parametric datasets), or one-way or two-way ANOVA as appropriate, with Bonferroni post-hoc tests. P-values less than 0.05 were considered statistically significant.

## Results

### Pattern of serum-induced proliferation is similar in HK-2 and ADPKD cells

**Fig. 1:**
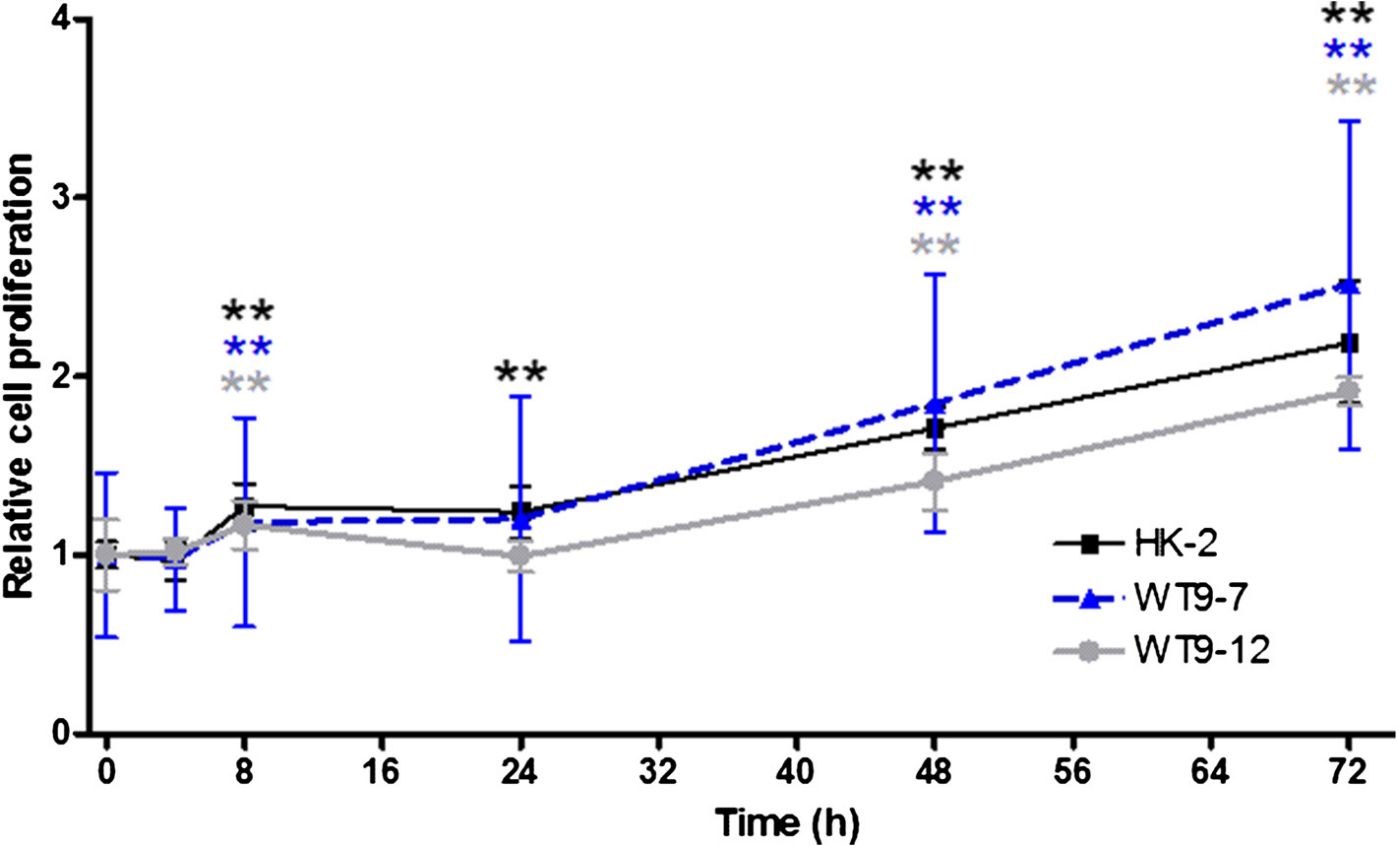
Proliferation of normal and ADPKD cells over a 72 h period. Serum-induced proliferation was assessed by BrdU assay in HK-2, WT9-7 and WT9-12 cells. Cell proliferation is expressed as the fold-change in absorbance over 0 h for the corresponding cell line. Data are expressed as mean ± SD from 2 experiments, with *n* = 6–8 per treatment group in each experiment. **p < 0.01 vs. 0 h for corresponding cell line

Serum-induced proliferation was assessed by a time-course BrdU assay of HK-2, WT9-7 and WT9-12 cells. In all three cell lines, an increase in proliferation was observed over time (Fig. 1)

### Onset of anti-proliferative effects of PDTC are delayed in ADPKD cells compared to HK-2 cells

Prior to assessing the effects of PDTC on serum-induced proliferation, we firstly assessed the compound’s cytotoxicity by MTT assay. This revealed a time- and dose-dependent effect of PDTC on the viability of HK-2, WT9-7 and WT9-12 cells (Table 1). At 24 h in all cell lines, viability was significantly decreased in the 50 μM PDTC group compared to the control. At 48 h, the lowest tested concentration (5 μM) decreased cell viability.

**Table 1:** Effect of PDTC on the percentage of viable HK-2, WT9-7 and WT9-12 cells, assessed by MTT assay after 6 h, 24 h and 48 h of treatment

	HK-2	WT9-7	WT9-12
6 h			
Control	100 ± 19.0	100 ± 10.2	100 ± 13.9
5 μM	99.6 ± 8.5	110 ± 26.8	103.0 ± 9.8
20 μM	98.8 ± 9.2	120.2 ± 13.1	97.1 ± 7.8
50 μM	76.8 ± 13.5	43.4 ± 9.3 **	87.6 ± 14.1
24 h			
Control	100 ± 15.0	100 ± 8.8	100 ± 12.6
5 μM	105.8 ± 7.7	85.2 ± 20.1	97.8 ± 14.3
20 μM	112.2 ± 7.6	103.6 ± 27.3	99.9 ± 9.1
50 μM	42.7 ± 2.8 **	17.1 ± 7.7 **	72.9 ± 5.9 **
48 h			
Control	100 ± 9.6	100 ± 13.3	100 ± 5.9
5 μM	76.9 ± 7.1 **	50.4 ± 11.7 **	75.9 ± 8.4 **
20 μM	90.3 ± 10.9	91.7 ± 24.0	82.1 ± 8.0 **
50 μM	19.3 ± 2.0 **	6.0 ± 0.5 **	51.7 ± 9.2 **

Data are expressed as mean ± SD from *n* = 8 wells. **p < 0.01 vs. untreated control for the same cell line at the corresponding timepoint

**Fig. 2:**
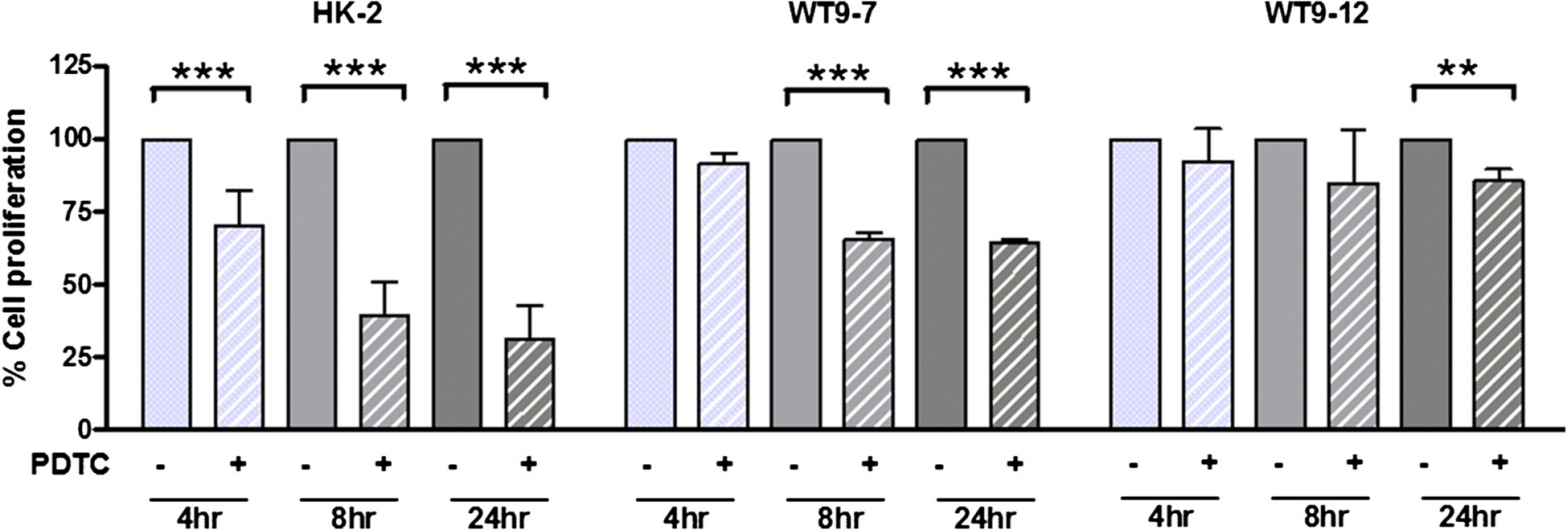
Effect of PDTC on proliferation in normal and ADPKD cells. HK-2, WT9-7 and WT9-12 cells were treated with 20 μM PDTC for 4, 8 and 24 h. Cell proliferation was calculated as the percentage change in absorbance, over the vehicle-treated group, for each experiment. Data as Mean + SD from *n* = 3 experiments. **p = 0.01, ***p < 0.001

Since cell viability was not significantly altered using 20 μM PDTC at 24 h (Table 1), this concentration and incubation period were selected for subsequent experiments. In HK-2 cells, PDTC treatment resulted in significant decreases in proliferation compared to untreated control after 4, 8 and 24 h of incubation (Fig. 2). In contrast, in WT9-7 cells PDTC significantly reduced proliferation only after 8 h and 24 h of treatment, and in WT9-12 cells an anti-proliferative effect was only observed at the 24 h timepoint.

**Fig. 3:**
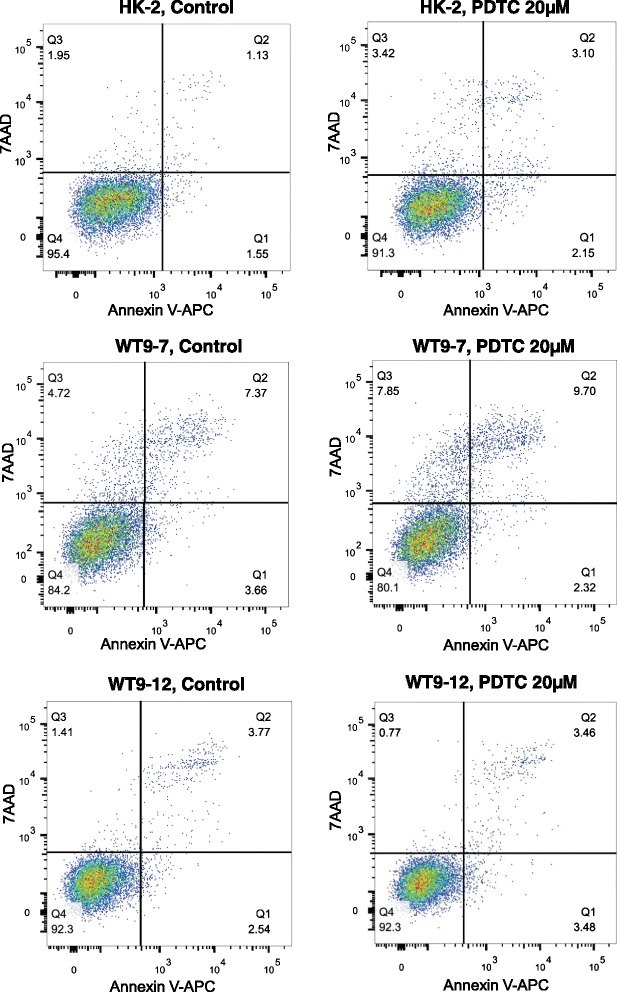
Effect of PDTC on apoptosis in normal and ADPKD cells. Representative FACS plots of Annexin V and 7AAD in HK-2, WT9-7 and WT9-12 cells

Flow cytometry for Annexin V found that 24 h incubation with 20 μM PDTC did not alter apoptosis in any cell line (*p* > 0.05, mean ± SD of *n* = 9 from two experiments, Table 2 and Fig. 3).

**Table 2:** Percentage of Annexin V positive cells

	Control	PDTC
HK-2	3.8 ± 1.5	5.2 ± 1.8
WT9-7	10.3 ± 3.9	11.2 ± 5.0
WT9-12	6.5 ± 2.0	7.2 ± 1.1

### TNF-α-induced NF-κB reporter activity is abrogated by PDTC in HK-2 but not in ADPKD cells

**Fig. 4:**
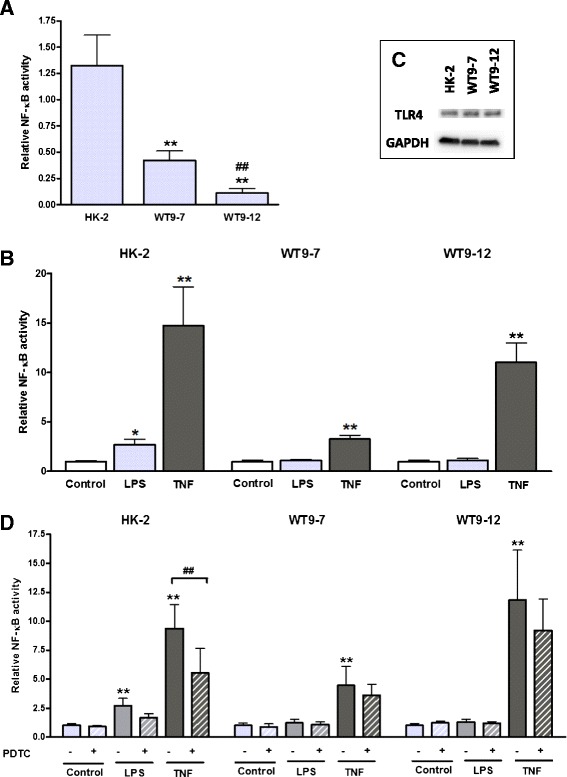
NF-κB-dependent luciferase reporter activity and TLR4 expression in normal and ADPKD cells. **a** Endogenous NF-κB activity in HK-2, WT9-7 and WT9-12 cells, 24 h post-transfection. **p < 0.01 vs. HK-2, ##*p* < 0.01 vs. 9–7 cells. Data are expressed as mean + SD from 3 experiments. **b** NF-κB activity was stimulated by 4 h of incubation with LPS (10 μg/mL) or TNF-α (20 ng/mL). NF-κB activity is expressed as the fold-change in luciferase/GFP over untreated control. *p < 0.05 vs. vehicle control for the corresponding cell line, **p < 0.01 vs. vehicle control for the corresponding cell line. Data are expressed as mean + SD from 3 experiments. **c** Expression of TLR4 in HK-2, WT9-7 and WT9-12 cells, determined by western blotting in whole cell lysates. **d** PDTC decreased TNF-α-induced NF-κB activity in HK-2 but not in WT9-7 or WT9-12 cells. Data are expressed as mean + SD from 2 experiments. **p < 0.01 vs. vehicle control for the corresponding cell line; ##*p* < 0.01

Endogenous NF-κB activity was assessed by luciferase reporter assay 24 h post-transfection and found to be lower in ADPKD cells compared with HK-2 cells (Fig. 4a). In addition, NF-κB activity was lower in WT9-12 cells compared to WT9-7. Given the low basal levels of NF-κB activity in ADPKD cells, we sought to determine the effects of classical NF-κB stimuli, namely LPS and TNF-α, on NF-κB activity in the three cell lines. In HK-2 cells, stimulation with LPS (10 μg/ml) for 4 h resulted in a three-fold increase in NF-κB activity over vehicle control, while TNF-α (20 ng/ml) caused a 15-fold increase in NF-κB activity (Fig. 4b). In contrast, LPS failed to induce a significant increase in luciferase NF-κB activity in WT9-7 and WT9-12 cells. However, the PKD cells were responsive to TNF-α, which increased NF-κB activity three-fold in WT9-7, and 11-fold in WT9-12 cells, over baseline. Western blotting demonstrated that all cells expressed TLR4 (a key receptor involved in mediating LPS-induced NF-κB signaling [38], Fig. 4c), suggesting that the lack of response to LPS in PKD cells was not due to an absence of this protein. Incubation of the cells with 20 μM PDTC abated TNF-α-induced NF-κB stimulation in HK-2 but not in WT9-7 or WT9-12 cells (Fig. 4d).

**Fig. 5:**
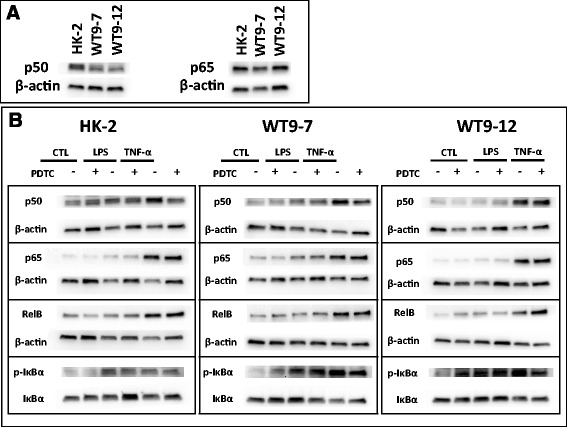
Expression of NF-κB proteins in nuclear extracts from normal and ADPKD cells. **a** Endogenous nuclear p50 and p65 expression. Representative blots from *n* = 3 experiments. **b** Representative western blots demonstrating the effects of LPS, TNF-α and PDTC on nuclear NF-κB p50, p65 and RelB, and cytosolic p-IκB levels

To confirm the results of the luciferase assay, western blotting for nuclear NF-κB proteins was performed. In contrast to the luciferase assay, immunoblotting indicated that basal nuclear expression of p50 and p65 was comparable between HK-2, WT9-7 and WT9-12 cells (p50 expression: HK-2, 1.0 ± 0.2; WT9-7, 1.4 ± 1.2; WT9-12, 0.9 ± 0.2, *p* > 0.05; p65 expression: HK-2, 1.0 ± 0.1; WT9-7, 2.2 ± 2.2; WT9-12, 1.2 ± 0.7, *p* > 0.05, Fig. 5a).

**Fig. 6:**
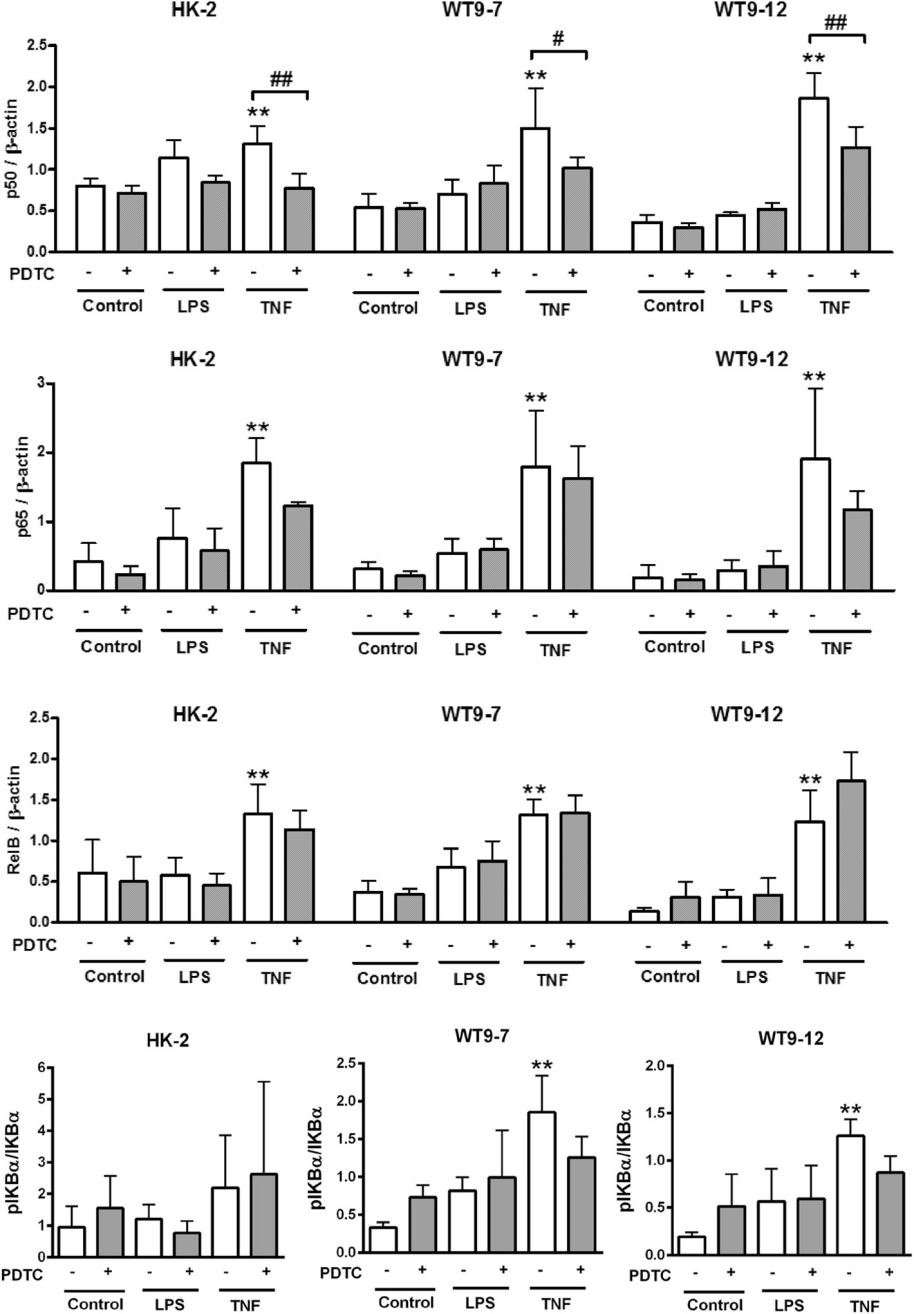
Densitometry quantification of western blot data. Data are expressed as mean + SD from *n* = 3 experiments. **p < 0.01 vs. vehicle-treated control; #*p* < 0.05; ##*p* <0.01

Nuclear levels of p50, p65 and RelB were up-regulated by 4 h of stimulation with TNF-α in all cell lines, but were not significantly altered by LPS (Figs. 5b and 6). PDTC decreased the TNF-α-induced increase of nuclear p50, but not of nuclear p65 or RelB, although there was a trend toward a reduction in p65 in HK-2 cells (*p* = 0.099). Cytoplasmic p-IκBα was increased with TNF-α stimulation in WT9-7 and WT9-12 cells but not in HK-2 cells. PDTC did not attenuate the TNF-α-induced increase in p-IκBα. No changes in IκBα were detected in any cell lines (*p* > 0.05, Additional file 1: Table S1).

### Classical NF-κB stimulants do not increase proliferation in either HK-2 or PKD cells

**Fig. 7:**
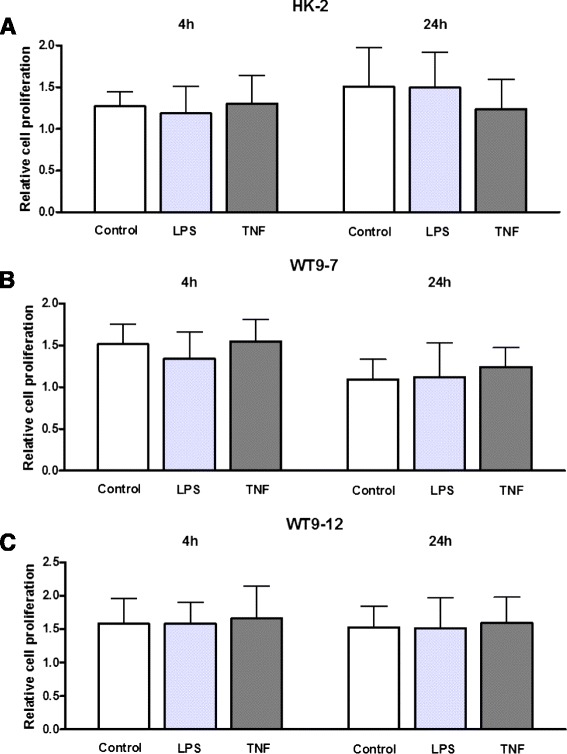
Effects of LPS and TNF-α on proliferation of normal and ADPKD cells. BrdU assay was performed in **a** HK-2, **b** WT9-7 and **c** WT9-12 cells following 4 h and 24 h of treatment with LPS (10 μg/ml) or TNF-α (20 ng/ml). Cell proliferation is expressed as the fold-change in absorbance over 0 h for the corresponding cell line. Data as mean + SD of *n* = 12 wells from 3 experiments

We then verified whether the effects of LPS and TNF-α on NF-κB activity could be associated with any effects on proliferation. BrdU assay demonstrated that compared to vehicle control, LPS (10ug/ml) and TNF-α (20 ng/ml) did not alter proliferation in any of the cell lines, at either 4 h or 24 h of treatment (Fig. 7).

## Discussion

We previously showed that chronic administration of PDTC to LPK rats decreased renal cyst growth but was not associated with changes in interstitial inflammation, fibrosis, cell proliferation or NF-κB activity [16]. However, since in this model cell proliferation is elevated in early stages (week 3) compared to later stages of life (week 12), [39] PDTC may have had early anti-proliferative effects that were not detectable at the final timepoint (week 11). Therefore, the primary objective of the current study was to determine, *in vitro*, whether inhibition of cell proliferation and NF-κB signaling can explain the cyst-inhibiting properties of PDTC. In addition, to extend upon our *in vivo* study which was conducted in a non-orthologous model of ARPKD, we used WT9-7 and WT9-12 cells, which possess mutated *PKD1* allele/s and truncated polycystin-1 protein, to model cellular function in ADPKD.

Cyst growth in PKD is associated with excessive proliferation of cyst-lining cells, and can be ameliorated by anti-proliferative agents [40, 41]. Polycystin-1-depleted renal cells exhibit accelerated proliferation compared to controls [42, 43], and since WT9-7 and WT9-12 cells have abnormal polycystin-1 expression [26], we hypothesized that proliferation rates would be higher in WT9 cells than in normal HK-2 cells. Instead, we found that HK-2, WT9-7 and WT9-12 cells have similar basal proliferation kinetics. This suggests that the *PKD1* mutation in and of itself does not lead to increased cell proliferation, and therefore extrinsic factors may be responsible. Our results contrast with those of Aguari *et al.,* which found increased proliferation rates in WT9-7 and WT9-12 cells relative to normal 4/5 cells [44]. Notably, the 4/5 control cells used by Aguari *et al.* were transformed by the same method as were the WT9-7 and WT9-12 cells (i.e. SV40 T antigen) [28, 44], whereas our control cell line, HK-2, was transformed by HPV16 E6/E7 [24]. Although we are not aware of any comparative studies of proliferation between these vectors, it is conceivable that immortalization may have altered the basal proliferative properties of the cell lines used.

The current study showed that PDTC reduced proliferation in ADPKD cells. This contrasts with our study in LPK rats, in which chronic PDTC administration diminished cyst growth without decreasing Ki67-positive cell proliferation [16], and may suggest that PDTC has immediate rather than long-term anti-proliferative effects. Interestingly, the anti-proliferative effects of PDTC occurred earlier in HK-2 compared to PKD cells. This may suggest that the *PKD1* mutation confers some resistance to the anti-proliferative effects of PDTC. The anti-proliferative effects of PDTC in HK-2 cells could be viewed as harmful for the healthy cell population in renal disease. However, it is worth noting that in our study, cells were cultured in sub-confluent conditions, which differ to the healthy kidney. Furthermore, it is known that susceptibility to drug cytotoxicity is decreased in cells grown at higher compared to lower densities [45, 46]. Therefore PDTC may not necessarily be detrimental under physiological conditions, in which the complete cell-cell contacts may confer some protection against the drug’s anti-proliferative effects.

There is evidence that the NF-κB system is up-regulated in experimental models of PKD [11, 12]. However, in our study, immunoblotting suggested that endogenous p50 and p65 levels were comparable between normal and PKD cells, and luciferase assay data indicated that basal NF-κB activity was in fact lower in PKD cells compared to normal cells. It is worth noting that whereas the luciferase assay measures DNA-binding activity of NF-κB proteins, immunoblotting only assesses the quantity of nuclear NF-κB proteins, and this may explain the differences between the results obtained by these two techniques. Our findings differ to those of Aguari *et al.,* which demonstrate that NF-κB nuclear translocation is elevated in WT9-7 and WT9-12 compared to 4/5 cells [44]. The differences between our findings may be explained by the use of differing NF-κB plasmids and immortalization techniques (as discussed above). It is also possible that the immortalization process itself can modulate basal NF-κB activity, and therefore future studies could verify this result by examining basal NF-κB activity in primary ADPKD cells.

We also sought to investigate the effects of classical NF-κB stimuli in immortalized ADPKD cells. Tumor necrosis factor-α directly induces cystogenesis in kidney explants [47] and regulates its own transcription via NF-κB in murine PKD cells [48, 49]. Our study found TNF-α to be a potent inducer of NF-κB in both normal and ADPKD cells, concurring with previous data from transformed cells overexpressing polycystin-1 [14]. In addition, we observed stimulus-specific induction of NF-κB in ADPKD cells, whereby both LPS and TNF-α up-regulated luciferase reporter NF-κB activity in HK-2 cells, while only TNF-α significantly up-regulated NF-κB in WT9 cells. Both LPS and TNF-α have been consistently identified in ADPKD cyst fluid [47, 50–52], thus it is unlikely that the lack of response to LPS is related to the availability of LPS in the cyst lumen. Since the lack of LPS-induced NF-κB stimulation is not due to an absence of TLR4 (a key receptor in the LPS-stimulated pro-inflammatory pathway [38]), other possible explanations for the discrepancy include defects in TLR4 or other proteins necessary for LPS binding, or differences in downstream signaling pathways. The discrepancy may also reflect differing roles of LPS and TNF-α in PKD etiology; TNF-α is known to induce cystogenesis *in vivo* [47], while it is unclear whether LPS can independently stimulate cyst growth in PKD [51, 53, 54]. Although LPS and TNF-α have previously been found to increase proliferation of renal cells *in vitro* [55, 56], neither stimulus altered proliferation in human PKD cells in this study, suggesting that the effects of these agents on NF-κB activation were not mediated through changes in cellular growth.

PDTC inhibits NF-κB activity by preventing proteasomal degradation of IκB, thereby retaining NF-κB proteins in the cytoplasm and obstructing their translocation to the nucleus [57]. PDTC decreased TNF-α-induced NF-κB luciferase reporter activity in HK-2 cells but not in WT9 cells, indicating that compared to normal cells, ADPKD cells may be less susceptible to NF-κB inhibition. In agreement with this, PDTC did not attenuate TNF-α-induced elevations in nuclear p65 or RelB or cytoplasmic p-IκBα, although it did decrease nuclear p50 expression in all cell lines. Notably, immunoblotting was performed on extracts obtained at a single timepoint (4 h following stimulation by LPS or TNF-α), whilst the luciferase assay measured cumulative NF-κB activity over the entire treatment period. Thus, nuclear p65 and RelB levels may have rapidly decreased early within the treatment period and restabilized by 4 h, whilst nuclear p50 levels may have decreased more steadily over time. It is known that p50 and p65 differ in their susceptibility to inhibition by IκB [58, 59], and particular IκB proteins inhibit certain dimer combinations more selectively than others (e.g. IκBα preferentially binds p65:p50 dimers over p65 homodimers) [60]. The reduction by PDTC in nuclear p50 but not p65 and RelB, may suggest that PDTC is more effective at preventing degradation of IκB proteins associated with p50, than of those binding the other NF-κB proteins. Given that p50 is structurally different to p65 and RelB (which possess transactivation domains), and that p50 homodimers can in fact repress transcription [10], future studies would benefit from characterizing the subunit composition of NF-κB complexes inhibited by PDTC.

In this study we used WT9-7 and WT9-12 cells, which are respectively heterozygous and homozygous for a *PKD1* mutant allele. Full-length polycystin-1 (the gene product of *PKD1*) is present in WT9-7 but not in WT9-12 cells [26]. Within the “two-hit hypothesis” of ADPKD (which proposes that for cystogenesis to occur, a cell must possess both an inherited germline and a somatically acquired *PKD1/2* mutation [61]), WT9-7 cells represent those cells possessing only the germline mutation, while WT9-12 cells represent those having acquired a second somatic hit. The hypothesis proposes that following the second somatic mutation, cells enter a hyperproliferative state and expand clonally [26, 61]. Whilst we did not find a difference in basal proliferation between WT9-7 and WT9-12 cells, the delayed anti-proliferative effect of PDTC on WT9-12 compared to WT9-7, might support the notion that loss of heterozygosity confers abnormal proliferative properties.

More recently, the “gene dosage” hypothesis has proposed that a second somatic mutation is not prerequisite for cystogenesis, but rather that cystogenesis commences if the amount of functional polycystin varies outside a certain range [27, 62, 63]. Compared to normal cells, WT9-7 cells are more resistant to the anti-proliferative and NF-κB-inhibiting properties of PDTC, and respond abnormally to fluid-shear stress [26]. Although it is possible that there is an undetected somatic mutation in WT9-7 cells [26], these observations may imply that levels of full-length polycystin-1 in WT9-7 cells are below the necessary “dosage” for normal cellular function [26]. Furthermore, compared to WT9-7 cells, WT9-12 cells had a delayed response to the anti-proliferative effects of PDTC (24 h vs. 8 h), and had lower basal NF-κB activity but a higher fold-increase in NF-κB following TNF-α stimulation. These data suggest that abnormalities in proliferation and NF-κB signaling may be correlated to the amount of full-length polycystin-1 expressed by a cell, although further studies are required to verify this trend and determine whether a causal relationship exists.

There were some limitations to this study. PDTC has pleiotropic properties including DNA damage [64, 65], anti-oxidant effects [66] and gene modulation [67], which were not examined in this study. Importantly, the results observed in WT9 cells do not necessarily explain the effects of PDTC in LPK rats, since the two models have dissimilar genetic mutations (*PKD1* vs. *NEK8*) [17, 25], and due to intrinsic differences between *in vitro* and *in vivo* models. Furthermore, the WT9 cells, which grow as a monolayer, cannot replicate the trans-epithelial fluid and ion transport processes that occur in three-dimensional renal cysts. Future studies could utilize collagen matrix cyst models to investigate whether PDTC inhibits cyst growth *in vitro*, and thereby determine whether TNF-α-induced cystogenesis is dependent on NF-κB [47]. Finally, the effects of the NF-κB stimuli were observed at single concentrations, and further work could verify whether varying concentrations of LPS and TNF-α differentially modify NF-κB activation in PKD cells.

## Conclusions

This study found that PDTC decreases the proliferation of immortalized human ADPKD cells, suggesting that this may be the mechanism of action by which the compound decreases cyst growth in experimental PKD [16]. Anti-proliferative effects of PDTC were delayed in ADPKD cells compared to normal cells. PDTC reduced TNF-α-stimulated NF-κB activity in HK-2 but not in ADPKD cells. Hence, in HK-2 cells there was an association between the reduction of proliferation and NF-κB activity, but no such association was observed in ADPKD cells (Table 3). Overall, this suggests that NF-κB functionality may be less critical for growth in ADPKD cells than in normal cells. It is worth noting that this study only identified an association between the effects of PDTC on proliferation and NF-κB activity, and further work is required to verify whether NF-κB-dependent proliferation is dysregulated in ADPKD cells. Future studies should also examine whether NF-κB stimulation can directly instigate cystogenesis and whether specific NF-κB inhibition is a viable strategy to abrogate cyst expansion in PKD.

**Table 3:** Summary of the main findings of this study

	HK-2	WT9-7	WT9-12
Pattern of serum-induced proliferation	Increased in a time-dependent manner	Same pattern as HK-2 cells	Same pattern as HK-2 cells
Effect of PDTC on proliferation	Reduced at 4-24 h	Reduced at 8-24 h	Reduced at 24 h
Endogenous NF-κB reporter activity	High	Moderate	Low
Effect of exogenous LPS on NF-κB reporter activity	Increased	No change	No change
Effect of exogenous TNF-α on NF-κB reporter activity	Increased	Increased	Increased
Effect of PDTC on TNF-α-induced NF-κB reporter activity	Reduced	No change	No change
Effect of PDTC on TNF-α-induced nuclear NF-κB protein levels	Reduced p50	Reduced p50	Reduced p50
Effect of exogenous LPS and TNF-α on proliferation at 4 h and 24 h	Same as unstimulated cells	Same as unstimulated cells	Same as unstimulated cells

## Additional file

Additional file 1:Table S1. Densitometry values for Western of cytoplasmic IκB (normalized using GAPDH). (DOC 29 kb)

## Abbreviations

7-AAD: 7-Aminoactinomycin D
ADPKD: Autosomal dominant polycystic kidney disease
APC: allophycocyanin
ARPKD: Autosomal recessive polycystic kidney disease
BrdU: Bromodeoxyuridine
CEC: Cystic epithelial cell
DMEM: Dulbecco’s Modified Eagle Medium
FACS: Fluorescent activated cell sorting
FBS: Fetal bovine serum
LPS: Lipopolysaccharide
GFP: Green fluorescent protein
hCMV: Human cytomegalovirus
MRI: Magnetic resonance imaging
NF-κB: Nuclear factor-κB
PBS: Phosphate buffered saline
PDTC: Pyrrolidine dithiocarbamate
PKD: Polycystic kidney disease
TLR4: Toll-like receptor 4
TNF-α: Tumor necrosis factor-α
