# Supplementary material for: Effects of pyrrolidine dithiocarbamate on proliferation and nuclear factor-κB activity in autosomal dominant polycystic kidney disease cells
Source: BMC Nephrol. 2015 Dec 15;16:212. doi: 10.1186/s12882-015-0193-3 (PMC4678764; doi:10.1186/s12882-015-0193-3)
Supplement: Additional file 1:Table S1. — Densitometry values for Western of cytoplasmic IκB (normalized using GAPDH). (DOC 29 kb) [file 12882_2015_193_MOESM1_ESM.doc]

**Additional file 1: Table S1.** Densitometry values for Western of cytoplasmic IκB (normalized using GAPDH)

|  | **CTL+V** | **CTL+P** | **LPS+V** | **LPS+P** | **TNF+V** | **TNF+P** |
| --- | --- | --- | --- | --- | --- | --- |
| **HK-2** | 0.73 ± 0.50 | 0.55 ± 0.23 | 1.17 ± 0.30 | 1.56 ± 0.59 | 0.56 ± 0.13 | 0.57 ± 0.27 |
| **WT9-7** | 2.76 ± 1.82 | 2.53 ± 1.19 | 2.54 ± 1.27 | 2.31 ± 1.57 | 1.81 ± 1.36 | 2.44 ± 1.64 |
| **WT9-12** | 0.94 ± 0.05 | 1.43 ± 0.63 | 1.79 ± 0.92 | 1.44 ± 0.29 | 1.06 ± 0.26 | 1.27 ± 0.57 |

Abbreviations: CTL, control; P, PDTC; V, vehicle. n=2-3 experiments.
